# Risk of myocardial infarction in patients with multiple sclerosis: a systematic review and meta-analysis

**DOI:** 10.1186/s12883-026-04882-w

**Published:** 2026-04-13

**Authors:** Shankar Biswas, Sindhu Vasireddy, Yashasvi Srivastava, Ayman Hamadttu

**Affiliations:** 1 Department of Internal Medicine, Ivano-Frankivsk National Medical University, Ivano-Frankivsk, Ukraine; 2 DM Neurology, Specialist Neurology NMC Specialty Hospital Abu Dhabi, Abu-Dhabi, United Arab Emirates; 3 Sudan University of Science and Technology, Khartoum, Sudan

**Keywords:** multiple sclerosis, myocardial infarction, cardiovascular disease, meta-analysis, systematic review, autoimmune disease, cardiovascular risk

## Abstract

**Background:**

Multiple sclerosis (MS) is associated with increased cardiovascular morbidity, yet the magnitude of myocardial infarction (MI) risk remains uncertain. With newer cohort studies and advances in meta-analytic methods, an updated evidence synthesis is warranted. We conducted a systematic review and meta-analysis to quantify MI risk in MS patients.

**Methods:**

We searched PubMed, Embase, and Scopus from inception through December 2025 for observational studies comparing MI risk between adults with MS and controls. Random-effects meta-analysis using restricted maximum likelihood estimation with Hartung-Knapp-Sidik-Jonkman adjustment was performed. Risk of bias was assessed using the Newcastle-Ottawa Scale, and certainty of evidence was evaluated using GRADE. Mendelian randomization evidence was synthesized separately.

**Results:**

Twenty cohorts from 18 studies were included. Seven cohorts (97,996 MS patients; 824,497 controls) contributed to the primary meta-analysis after excluding one study due to overlapping populations. MS patients had significantly increased MI risk (RR 1.70, 95% CI: 1.57–1.85; *p* < 0.0001) with moderate heterogeneity (I²=40.3%). The 95% prediction interval (1.60–1.81) indicated consistently elevated risk across settings. Secondary analyses showed increased stroke risk (RR 1.66, 95% CI: 1.01–2.73). Subgroup analyses revealed no significant differences by geographic region (*p* = 0.066), adjustment status (*p* = 0.45), or effect measure type (*p* = 0.79). Leave-one-out sensitivity analysis confirmed robust findings (RR range: 1.69–1.76). Mendelian randomization evidence showed a weak genetic association (OR 1.03, 95% CI: 1.00-1.06). GRADE certainty was very low.

**Conclusions:**

MS patients have a 70% increased risk of MI compared to individuals without MS. This risk elevation, comparable to other autoimmune conditions with established cardiovascular screening guidelines, supports the need for enhanced cardiovascular surveillance in MS populations.

**Supplementary Information:**

The online version contains supplementary material available at 10.1186/s12883-026-04882-w.

## Introduction

Multiple sclerosis (MS) is a chronic inflammatory demyelinating disease of the central nervous system affecting approximately 2.8 million people worldwide, with a global prevalence of 35.9 per 100,000 population [1]. The Global Burden of Disease Study estimated 2.22 million prevalent cases and 1.15 million disability-adjusted life years (DALYs) attributable to MS, with the highest disease burden observed in North America and Western Europe [2]. Beyond the well-characterized neurological disability, there is growing recognition that individuals with MS face substantially elevated risks of systemic comorbidities, including migraine (OR 2.02, 95% CI: 1.14–3.57) [3], depression, and particularly cardiovascular disease (CVD).

Cardiovascular comorbidity has emerged as a significant contributor to morbidity and mortality in the MS population. A large UK cohort study demonstrated that MS patients have a 28% increased hazard of acute coronary syndrome (HR 1.28, 95% CI: 1.09–1.51), 59% increased cerebrovascular disease risk, and 47% elevated cardiovascular mortality compared to matched controls [4]. The burden of traditional cardiovascular risk factors, including hypertension (18.6%) and hypercholesterolemia (10.9%), is substantial in MS populations, and vascular comorbidities have been associated with accelerated disability progression [5].

Several systematic reviews and meta-analyses have attempted to quantify the association between MS and cardiovascular outcomes. Marrie et al. conducted a foundational systematic review in 2015 documenting increased ischemic heart disease and stroke risk across well-designed studies [6]. More recently, Rapp et al. reported a pooled hazard ratio of 1.6 (95% CI: 1.2-2.0) for myocardial infarction in MS patients based on nine longitudinal studies, though with considerable heterogeneity (I²=86%) [7]. Tavallaei et al. reported MI prevalence of 1.7% in MS with a pooled odds ratio of 1.41 [8]. Most recently, Ketata and Ellouz conducted a comprehensive meta-analysis examining vascular disease incidence and mortality in MS, reporting an increased incidence rate ratio for myocardial infarction (IRR 1.4, 95% CI: 1.0-1.9), with subgroup analysis suggesting the highest risk within the first 10 years following MS diagnosis [9]. Additionally, Stefanou et al. reported a 2.55-fold increased stroke risk in MS patients [10], further supporting the broader pattern of elevated cerebrovascular risk in this population.

The pathophysiological mechanisms underlying increased cardiovascular risk in MS are multifactorial. Chronic systemic inflammation, mediated by Th1, Th17, and CD8 + T cell pathways, may promote atherosclerosis and endothelial dysfunction [11]. Autonomic dysfunction, affecting up to two-thirds of MS patients, can contribute to cardiac arrhythmias and blood pressure dysregulation. Furthermore, subclinical cardiac dysfunction with impaired biventricular function has been demonstrated in MS patients independent of traditional vascular risk factors, suggesting intrinsic myocardial involvement [12]. Disease-modifying therapies, particularly fingolimod and anti-CD20 agents, may also confer additional cardiovascular effects.

Beyond observational evidence, Mendelian randomization (MR) studies provide genetic data relevant to the MS-cardiovascular relationship. Yang et al. reported that genetic liability to MS was associated with a small increased risk of myocardial infarction (OR 1.03, 95% CI: 1.00-1.06, *p* = 0.01) and coronary artery disease using 68 genetic instruments [13]. While sensitivity analyses supported the validity of causal inference, the small magnitude of these genetic effects warrants cautious interpretation. Given the availability of newer cohort studies published between 2020 and 2025 and advances in meta-analytic methodology, we conducted an updated systematic review and meta-analysis to quantify the risk of myocardial infarction in MS patients compared to the general population.

## Methods

### Protocol registration

This systematic review was registered with PROSPERO (CRD420261276986). The protocol was registered prior to data extraction and analysis; however, registration occurred after the initial literature search was conducted, which represents a limitation.

### Search strategy and information sources

We conducted a systematic review following the Preferred Reporting Items for Systematic Reviews and Meta-Analyses (PRISMA) 2020 guidelines [14] and the Meta-analysis Of Observational Studies in Epidemiology (MOOSE) guidelines [15]. We searched PubMed, Embase, and Scopus from inception through December 2025. The search strategy combined terms for multiple sclerosis (“multiple sclerosis” OR “MS” OR “demyelinating disease”) AND cardiovascular outcomes (“myocardial infarction” OR “heart attack” OR “acute coronary syndrome” OR “ischemic heart disease” OR “cardiovascular disease” OR “stroke” OR “cerebrovascular”). We also manually searched reference lists of included studies and relevant systematic reviews to identify additional eligible studies.

### Eligibility criteria

Studies were included if they: (1) were observational cohort studies (prospective or retrospective) or case-control studies; (2) included adult patients (≥ 18 years) with a confirmed diagnosis of MS; (3) included a comparator group of individuals without MS; (4) reported myocardial infarction as an outcome with quantifiable effect estimates (hazard ratio [HR], incidence rate ratio [IRR], odds ratio [OR], or relative risk [RR]) with 95% confidence intervals (CIs) or sufficient data to calculate these; and (5) were published in English in peer-reviewed journals.

Studies were excluded if they: (1) were case reports, case series, editorials, reviews, or conference abstracts without full-text availability; (2) examined only MS patients without a control group; (3) did not report MI-specific outcomes (i.e., only composite cardiovascular endpoints); (4) had overlapping populations with larger or more recent studies; or (5) were Mendelian randomization studies (analyzed separately as supporting evidence).

### Data extraction

Two reviewers independently extracted data using a standardized form. Discrepancies were resolved through consensus. The following data were extracted: study identification (first author, publication year, country), study design, data source, study period, sample size (MS and control groups), demographic characteristics (age, sex distribution), MS diagnostic criteria, outcome definitions (ICD codes where available), follow-up duration, effect estimates with 95% CIs, and adjustment variables. For studies reporting multiple effect estimates, we preferentially extracted fully adjusted estimates. When studies reported outcomes at multiple timepoints, we extracted the overall or longest follow-up estimate.

### Risk of bias assessment

We assessed risk of bias using the Newcastle-Ottawa Scale (NOS) for cohort studies [16], which evaluates three domains: selection (4 items, maximum 4 stars), comparability (1 item, maximum 2 stars), and outcome (3 items, maximum 3 stars). For the comparability domain, studies received one star for adjustment for age and/or sex, and a second star for adjustment for additional cardiovascular risk factors (diabetes, hypertension, hyperlipidemia, smoking, or BMI). Studies scoring 7–9 stars were classified as low risk, 4–6 as moderate risk, and 0–3 as high risk. Two reviewers independently assessed each study, with disagreements resolved by discussion until consensus was reached. Methodological guidance on risk-of-bias assessment in observational meta-analyses from the Cochrane Handbook and MOOSE guidelines was followed [15].

### Statistical analysis

We performed random-effects meta-analysis using the restricted maximum likelihood (REML) estimator for between-study variance (τ²). Effect estimates (hazard ratios, incidence rate ratios, and event rate ratios) were natural log-transformed for analysis, and standard errors were derived from the reported 95% confidence intervals using the formula: SE = (ln(upper CI) - ln(lower CI)) / (2 × 1.96). These measures were pooled as relative risks given the rare outcome assumption, which holds when event rates are below approximately 10% [Cochrane Handbook for Systematic Reviews of Interventions, Chap. 10]. Odds ratios from prevalence studies were analyzed separately. We applied the Hartung-Knapp-Sidik-Jonkman adjustment for the random-effects model confidence intervals to account for uncertainty in τ² estimation, which provides more appropriate coverage particularly with small numbers of studies [17].

Heterogeneity was assessed using Cochran’s Q statistic (significance threshold *p* < 0.10), I² statistic (interpreted as low [< 25%], moderate [25–50%], substantial [50–75%], or considerable [> 75%]), and the 95% prediction interval to estimate the range of true effects expected in future studies conducted in similar settings.

We conducted pre-specified subgroup analyses by: (1) geographic region (North America, Europe, Asia-Pacific); (2) effect measure type (HR, IRR, ERR); and (3) adjustment status (adjusted vs. unadjusted estimates). Subgroup differences were tested using the Q-test for interaction.

Sensitivity analyses included: (1) leave-one-out analysis to assess the influence of individual studies; (2) exclusion of studies with overlapping populations; and (3) assessment of potential outliers.

Publication bias was assessed visually using funnel plots. Egger’s test was planned if ≥ 10 studies were available; however, this threshold was not met, and we note that funnel plot asymmetry tests are unreliable with fewer than ten studies. We performed trim-and-fill analysis to estimate the impact of potentially missing studies.

We assessed the certainty of evidence using the Grading of Recommendations Assessment, Development and Evaluation (GRADE) approach [18], considering risk of bias, inconsistency, indirectness, imprecision, and publication bias, with explicit justification for each domain rating.

Mendelian randomization (MR) studies were synthesized narratively as supporting evidence for potential causal relationships. All analyses were performed in R version 4.5.1 using the ‘meta’ (version 8.2-1) and ‘metafor’ (version 4.8-0) packages. Statistical significance was set at *p* < 0.05 (two-tailed).

## Results

### Study selection

**Fig. 1:**
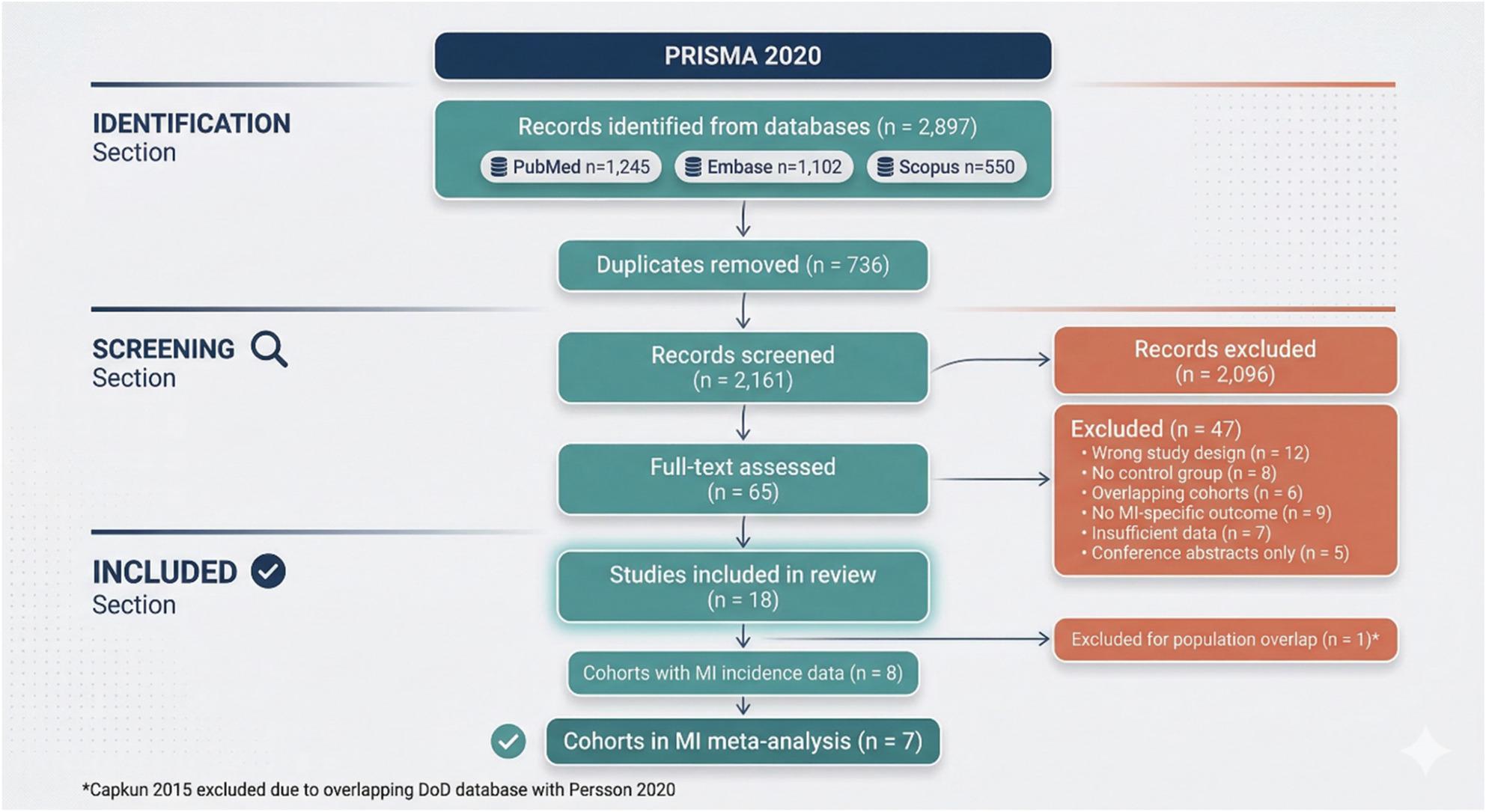
PRISMA flow diagram of study selection. Study selection conducted following PRISMA 2020 guidelines across three databases (PubMed n=1,245; Embase n=1,102; Scopus n=550), yielding 2,897 records; after removal of 736 duplicates, 2,161 records were screened and 65 full-text articles assessed. Of 18 studies (20 cohorts) meeting eligibility criteria, one cohort (Capkun 2015) was subsequently excluded from the primary meta-analysis due to population overlap with Persson 2020 DOD, leaving 7 cohorts for the primary analysis

The systematic search identified 2,897 records from PubMed (*n* = 1,245), Embase (*n* = 1,102), and Scopus (*n* = 550). After removing 736 duplicates, 2,161 records were screened by title and abstract, of which 2,096 were excluded. Sixty-five full-text articles were assessed for eligibility. Studies were excluded for the following reasons: wrong study design (*n* = 12), no control group (*n* = 8), overlapping cohorts (*n* = 6), no MI-specific outcome (*n* = 9), insufficient data (*n* = 7), and conference abstracts only (*n* = 5). Ultimately, 20 unique cohorts from 18 studies were included in the systematic review. After excluding one cohort (Capkun 2015) due to overlapping population with Persson 2020 DOD (both utilized the US Department of Defense database), 7 cohorts contributed to the primary meta-analysis of MI incidence (Fig. 1).

### Study characteristics

The characteristics of included studies are summarized in Table 1. Studies were published between 2008 and 2025 and conducted across North America (Allen 2008 [19]; Capkun 2014 [20], 2015 [21]; Persson 2020 US-DOD [22]; Marrie 2019 [23]; Franklin 2014 [24]; Kresa-Reahl 2017 [25]; Herial 2012 [26]; Goodman 2014 [27]), Europe (Christiansen 2010 [28]; Castelo-Branco 2020 [29]; Persson 2020 UK-CPRD [22]; Chou 2020 [30]; Benjaminsen 2021 [31]; Cabreira 2020 [32]; Bezzini 2022 [33]; Giallafos 2016 [34]), and Asia-Pacific (Cho 2022 [35]). Additional studies included Lo 2021 [36] from Australia and Hemida 2025 [37] reporting US mortality trends. Study designs included retrospective cohort, prospective cohort, cross-sectional, and case-control studies.

**Table 1:** Characteristics of Included Studies

Study	Country	Design	Data Source	Period	MS (*n*)	Controls (*n*)	NOS
Allen 2008 [19]	USA	Case-control	SPARCS	1988–2002	9,949	19,898	8
Christiansen 2010 [28]	Denmark	Cohort	DNRP	1977–2006	13,963	66,407	8
Herial 2012 [26]	USA	Cross-sectional	NIS	2006	20,843	6,590,420	6
Capkun 2014 [20]	USA	Cross-sectional	MarketScan	2006–2012	49,231	492,310	5
Franklin 2014 [24]	USA	Cross-sectional	NIS	2006–2010	136,542	—	4
Goodman 2014 [27]	USA	Cross-sectional	EHR CLIMB	NR	3,010	—	5
Capkun 2015 [21] ‡	USA	Cohort	DoD-NDI	2006–2011	15,684	78,420	6
Giallafos 2016 [34]	Greece	Cross-sectional	Single center	NR	183	—	4
Kresa-Reahl 2017 [25]	USA	Cross-sectional	IMS Health	2011–2015	66,616	66,616	6
Marrie 2019 [23]	Canada	Cohort	BC+Manitoba	1984–2016	14,565	72,825	9
Castelo-Branco 2020 [29]	Sweden	Cohort	NPR	2008–2016	6,602	61,828	8
Chou 2020 [30]	UK	Cohort	CPRD	1997–2006	2,526	9,980	8
Cabreira 2020 [32]	Portugal	Cohort	Hospital records	2009–2015	106	—	6
Persson 2020 (DOD) [22] †	USA	Cohort	US-DOD	2004–2017	6,406	66,281	7
Persson 2020 (CPRD) [22] †	UK	Cohort	UK-CPRD	2001–2016	5,726	57,331	7
Benjaminsen 2021 [31]	Norway	Cross-sectional	NPR	2008–2017	637	—	8
Lo 2021 [36]	Australia	Cross-sectional	AMSLS	2016	1,518	17,733,300	9
Bezzini 2022 [33]	Italy	Cross-sectional	Tuscan Admin	2019	8,274	2,917,181	7
Cho 2022 [35]	South Korea	Cohort	KNHIS	2010–2017	1,503	7,515	7
Hemida 2025 [37]	USA	Descriptive	CDC WONDER	1999–2023	53,903	—	7

Twenty cohorts from 18 studies published between 2008 and 2025, conducted across North America, Europe, and Asia-Pacific using national registries, administrative claims databases, and electronic health records.

*Abbreviations SPARCS* Statewide Planning and Research Cooperative System, *DNRP* Danish National Registry of Patients, *NIS* Nationwide Inpatient Sample, *DoD* Department of Defense, *NDI* National Death Index, *NPR* National Patient Register, *KNHIS* Korean National Health Insurance Service, *CPRD* Clinical Practice Research Datalink, *BC* British Columbia, *AMSLS* Australian MS Longitudinal Study, *NOS* Newcastle-Ottawa Scale, *NR* not reported

*— *no control group

†Persson 2020 reported two independent cohorts from different databases (1 study, 2 cohorts)

‡Capkun 2015 excluded from primary meta-analysis due to overlapping population with Persson 2020 DOD (both used US Department of Defense database)

Data sources included national health registries, administrative claims databases, and electronic health records. The mean/median age of MS patients ranged from 40.9 to 57.3 years where reported, and the proportion of females ranged from 53.0% to 79.6%, consistent with the known female predominance of MS.

### Primary outcome: myocardial infarction risk

**Fig. 2:**
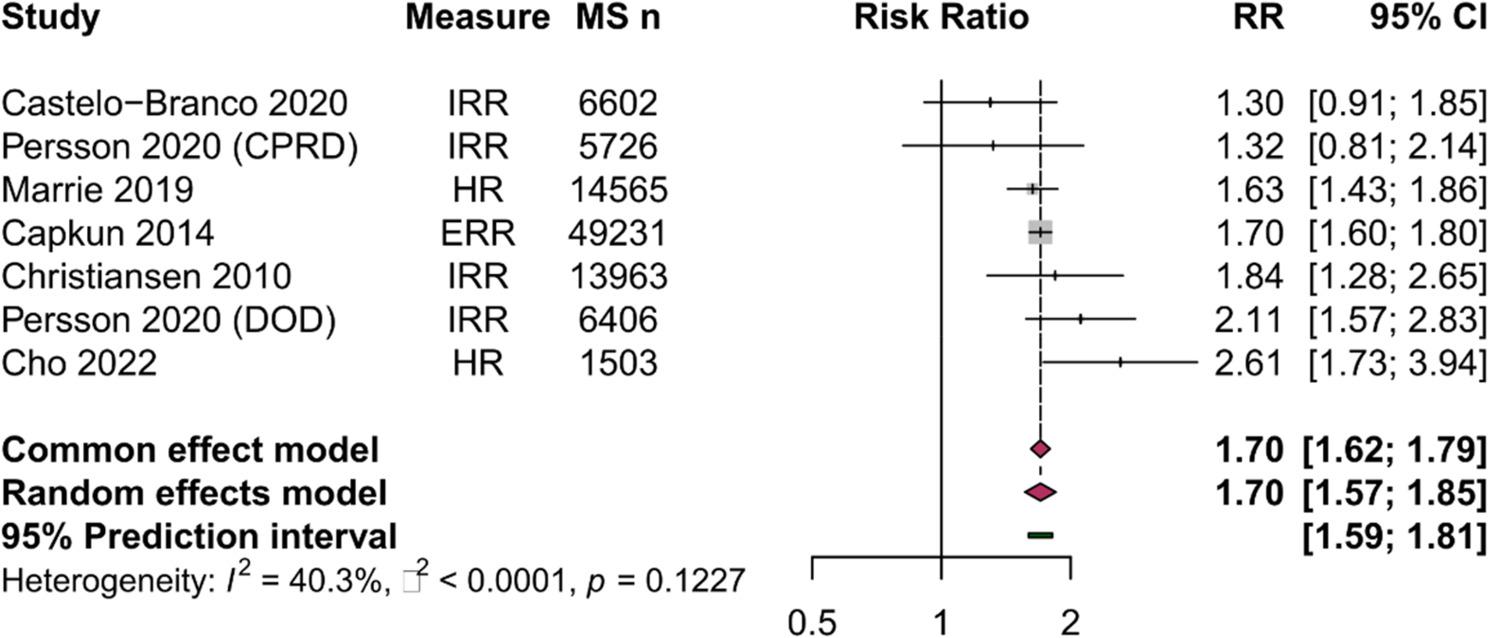
Forest plot of myocardial infarction risk in MS vs controls (primary meta-analysis). Individual study estimates (squares, sized proportional to weight) and pooled random-effects estimate (diamond) displayed on a logarithmic scale. Horizontal lines represent 95% confidence intervals; the vertical dashed line indicates the null value (RR=1.0). Pooled RR 1.70 (95% CI: 1.57–1.85; p<0.0001); moderate heterogeneity (I²=40.3%, p=0.12); 95% prediction interval 1.60–1.81, indicating consistently elevated MI risk expected across future study settings

Seven cohorts (97,996 MS patients; 824,497 controls) reported incidence-based effect estimates (HR, IRR, or ERR) for MI and were included in the primary meta-analysis [20, 22, 23, 28, 29, 35]. One study (Capkun 2015 [21]) was excluded due to overlapping population with Persson 2020 DOD, as both utilized the US Department of Defense database. The pooled relative risk of MI in MS patients compared with controls was 1.70 (95% CI: 1.57–1.85; *p* < 0.0001), indicating a 70% increased risk of MI (Fig. 2; Table 2).

**Table 2:** Primary Meta-Analysis Results: Myocardial Infarction Risk in MS

Study	Effect Measure	MS Events	Control Events	MS Total	Control Total	RR (95% CI)	Weight (%)
Capkun 2014 [20]	ERR	NR	NR	49,231	492,310	1.70 (1.60–1.80)	75.6
Castelo-Branco 2020 [29]	IRR	35	252	6,602	61,828	1.30 (0.91–1.85)	2.1
Cho 2022 [35]	HR	35	68	1,503	7,515	2.61 (1.73–3.94)	1.5
Christiansen 2010 [28]	IRR	30	78	13,963	66,407	1.84 (1.28–2.65)	2.0
Marrie 2019 [23]	HR	281	1,346	14,565	72,825	1.63 (1.43–1.87)	14.6
Persson 2020 [22] (CPRD)	IRR	NR	NR	5,726	57,331	1.32 (0.81–2.14)	1.1
Persson 2020 [22] (DOD)	IRR	NR	NR	6,406	66,281	2.11 (1.58–2.83)	3.1
Pooled (Random Effects)				**97**,**996**	**824**,**497**	**1.70 (1.57–1.85)**	**100**

Pooled relative risk derived from random-effects meta-analysis using restricted maximum likelihood estimation with Hartung-Knapp-Sidik-Jonkman confidence interval adjustment; hazard ratios, incidence rate ratios, and event rate ratios were pooled as relative risks under the rare outcome assumption (MI event rates < 5% across included studies). Study weights reflect the inverse-variance contribution under the random-effects model

*ERR* event rate ratio, *IRR *incidence rate ratio, *HR* hazard ratio,* NR *not reported

Bold text indicates pooled summary estimate from random-effects meta-analysis

Moderate heterogeneity was observed (I² = 40.3%, 95% CI: 0.0-74.9%; τ² < 0.0001; Q = 10.05, df = 6, *p* = 0.12). The 95% prediction interval ranged from 1.60 to 1.81, indicating that future studies would be expected to show increased MI risk across diverse settings.

Two studies reported prevalence-based odds ratios for MI history [19, 26]. The pooled OR was 0.73 (95% CI: 0.64–0.82; *p* < 0.0001), derived using fixed-effects meta-analysis given the absence of heterogeneity (I²=0.0%) across these two prevalence studies, suggesting lower MI prevalence in hospitalized MS patients compared to controls. This apparent paradox, lower prevalence but higher incidence, likely reflects survival bias (MS patients with prior MI may have higher mortality and thus be underrepresented in prevalent cases), differential healthcare utilization patterns, or the younger age structure of hospitalized MS populations compared to general hospital controls. These cross-sectional studies provide complementary descriptive information but were not combined with incidence estimates given the fundamental difference in study design and outcome timing.

### Secondary outcomes

**Fig. 3:**
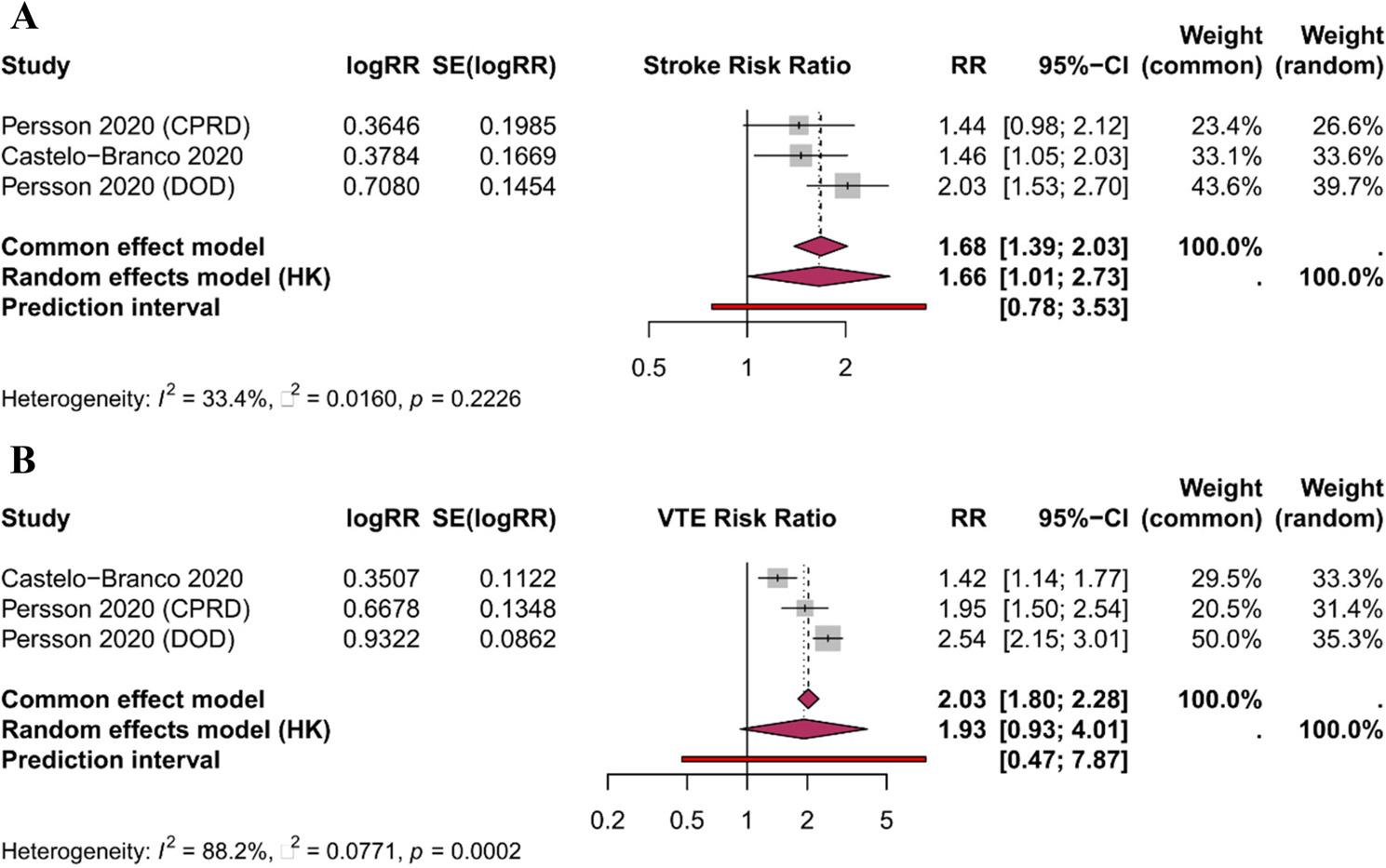
Secondary outcomes: **A** stroke risk; **B** VTE risk in MS vs controls. Forest plots comparing stroke (Panel **A**) and venous thromboembolism (Panel **B**) risk between MS patients and controls across 3 cohort studies per outcome (n = 18,734 MS patients; 185,440 controls). Panel **A**: Stroke risk was significantly elevated (RR 1.66, 95% CI: 1.01-2.73; p = 0.049; I² = 33.4%; prediction interval 0.78-3.53). Panel **B**: VTE risk showed non-significant near-doubling (RR 1.93, 95% CI: 0.93-4.01; p = 0.061; I² = 88.2%; prediction interval 0.47-7.87). Random-effects meta-analysis with Hartung-Knapp-Sidik-Jonkman adjustment. Abbreviations: RR, relative risk; CI, confidence interval; VTE, venous thromboembolism; MS, multiple sclerosis; logRR, natural logarithm of relative risk; SE, standard error; HK, Hartung-Knapp adjustment applied

- *Stroke*: Three cohorts reported stroke risk, with a pooled RR of 1.66 (95% CI: 1.01–2.73; *p* = 0.049; I² = 33.4%), indicating a 66% increased risk [22, 29] (Fig. 3A; Supplementary Table S2).
- *Venous Thromboembolism*: Three cohorts reported VTE risk, with a pooled RR of 1.93 (95% CI: 0.93–4.01; *p* = 0.061; I² = 88.2%), suggesting a near-doubling of risk that did not reach statistical significance due to high heterogeneity [22, 29] (Fig. 3B).
- *Heart Failure and Cardiovascular Mortality*: Insufficient studies (*n* < 2) with comparable effect measures precluded meta-analysis for these outcomes.

### Subgroup analyses

**Fig. 4:**
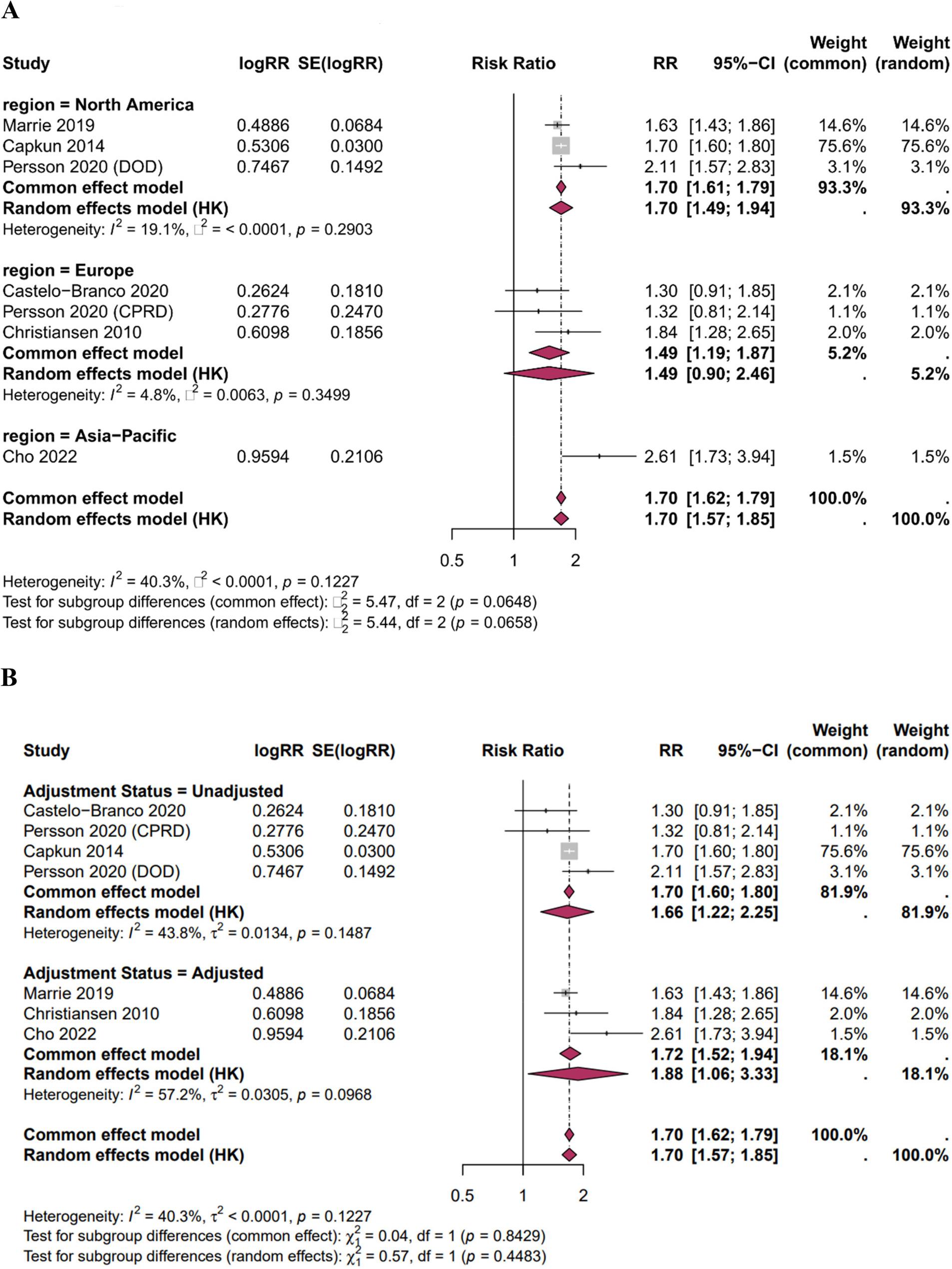
**A** Subgroup analysis of MI risk by geographic region. Forest plot displaying pooled relative risk estimates stratified by geographic region: North America (k=3; RR 1.70, 95% CI: 1.49–1.94), Europe (k=3; RR 1.49, 95% CI: 0.90–2.46), and Asia-Pacific (k=1; RR 2.61, 95% CI: 1.73–3.94). The test for subgroup differences did not reach statistical significance (p=0.066), suggesting a trend toward regional variation that warrants cautious interpretation given the limited number of studies per subgroup. **B** Adjusted vs unadjusted MI forest plot. Abbreviations: RR, risk ratio; CI, confidence interval; SE, standard error; HK, Hartung-Knapp variance correction; I², inconsistency statistic; τ², between-study variance; df, degrees of freedom; DOD, Department of Defense; CPRD, Clinical Practice Research Datalink. Notes: Subgroup analysis stratified by adjustment status (unadjusted vs. adjusted). Both common effect and random effects models (Hartung-Knapp method) are presented; moderate heterogeneity was observed in the adjusted subgroup (I² = 57.2%). No statistically significant subgroup differences were detected (common effect: p = 0.8429; random effects: p = 0.4483)

Subgroup analyses for MI risk are presented in Table 3; Fig. 4A-B.

- *Geographic Region* (Fig. 4A): The pooled RR was 1.70 (95% CI: 1.49–1.94) for North American studies (k = 3), 1.49 (95% CI: 0.90–2.46) for European studies (k = 3), and 2.61 (95% CI: 1.73–3.94) for the single Asia-Pacific study. The test for subgroup differences did not reach significance (*p* = 0.066).
- *Effect Measure Type* (Supplementary Figure S1): Pooled estimates were consistent across HR-based studies (k = 2; RR = 1.98, 95% CI: 0.10–37.50), IRR-based studies (k = 4; RR = 1.65, 95% CI: 1.12–2.45), and the ERR-based study (k = 1; RR = 1.70, 95% CI: 1.60–1.80), with no significant subgroup difference (*p* = 0.79). The wide confidence interval for HR studies reflects the Hartung-Knapp adjustment behavior with only two studies.
- *Adjustment Status* (Fig. 4B): Studies reporting adjusted estimates (k = 3; RR = 1.88, 95% CI: 1.06–3.33) showed similar results to unadjusted estimates (k = 4; RR = 1.66, 95% CI: 1.22–2.25), with no significant difference (*p* = 0.45). This suggests that confounding by traditional cardiovascular risk factors does not fully explain the increased MI risk in MS.

**Table 3:** Subgroup Analyses for MI Risk

Subgroup	k	Pooled RR (95% CI)	I² (%)	*p* for Interaction
Overall	7	1.70 (1.57–1.85)	40.3	-
Geographic Region				0.066
North America	3	1.70 (1.49–1.94)	19.1	
Europe	3	1.49 (0.90–2.46)	4.8	
Asia-Pacific	1	2.61 (1.73–3.94)	-	
Effect Measure				0.79
HR	2	1.98 (0.10–37.50)*	77.9	
IRR	4	1.65 (1.12–2.45)	45.7	
ERR	1	1.70 (1.60–1.80)	-	
Adjustment				0.45
Adjusted	3	1.88 (1.06–3.33)	57.2	
Unadjusted	4	1.66 (1.22–2.25)	43.8	

All subgroup comparisons tested using the Q-test for interaction

*k* number of cohorts, *HR* hazard ratio, *IRR* incidence rate ratio, *ERR* event rate ratio

*p*-values > 0.05 indicate no statistically significant subgroup difference

*The wide confidence interval for HR studies (95% CI: 0.10–37.50) is a known behavior of the Hartung-Knapp adjustment when only two studies are available (k = 2) and should be interpreted with caution rather than as evidence of heterogeneity. The single Asia-Pacific study precluded heterogeneity estimation for that subgroup.

### Sensitivity analyses

Leave-one-out analysis demonstrated robust results, with pooled RR ranging from 1.69 to 1.76 when individual studies were sequentially excluded (Supplementary Figure S4). No single study disproportionately influenced the overall estimate. Removing the largest study (Capkun 2014) modestly increased heterogeneity (I² from 40.3% to 50.2%) while maintaining a significant pooled estimate (RR = 1.75, 95% CI: 1.35–2.25).

### Publication bias

Visual inspection of the funnel plot showed reasonable symmetry (Supplementary Figure S2). Egger’s test was not performed due to insufficient studies (k < 10), as funnel plot asymmetry tests are unreliable with fewer than ten studies. Trim-and-fill analysis imputed zero missing studies, and the adjusted estimate remained unchanged (RR = 1.70, 95% CI: 1.57–1.85), suggesting no evidence of publication bias.

### Risk of bias assessment

Risk of bias assessment results are shown in Supplementary Table S3. Of 20 cohorts, 12 (60%) were rated as low risk of bias (NOS 7–9), 7 (35%) as moderate risk (NOS 4–6), and 1 (5%) as high risk (NOS 0–3). The mean NOS score was 6.8 out of 9. Common limitations included incomplete adjustment for confounders and reliance on administrative codes for outcome ascertainment without validation.

### Mendelian randomization evidence

One MR study [13] provided genetic evidence examining the relationship between MS liability and cardiovascular outcomes (Supplementary Table S4). Genetic liability to MS was associated with a small increased risk of coronary artery disease (OR = 1.02, 95% CI: 1.00-1.04; *p* = 0.03) and MI specifically (OR = 1.03, 95% CI: 1.00-1.06; *p* = 0.01) using inverse-variance weighted MR with 68 genetic instruments. While pleiotropy tests were non-significant (*p* > 0.10), the small magnitude of these genetic effects (2–3% increased risk) contrasts with the substantially larger observational estimate (70% increased risk), suggesting that unmeasured confounding or other biases may partly explain the observational association. These MR findings provide suggestive rather than definitive evidence for a causal relationship.

### Certainty of evidence

The GRADE assessment indicated low certainty evidence for the association between MS and MI risk (Table 4). Starting from low certainty (observational studies), evidence was downgraded for risk of bias (some studies lacked complete confounder adjustment). No downgrade was applied for inconsistency given the moderate heterogeneity (I² = 40.3%) with consistent direction across all studies, nor for publication bias given trim-and-fill analysis imputed zero missing studies. No upgrading factors were applicable given the observational design and modest effect magnitude.

**Table 4:** GRADE Certainty Assessment

Domain	Assessment	Rationale	Downgrade
Risk of Bias	Serious concerns	Some studies lacked complete confounder adjustment; reliance on administrative codes	-1
Inconsistency	No serious concerns	Moderate heterogeneity (I² = 40.3%); direction consistent across all studies; narrow prediction interval (1.60–1.81)	0
Indirectness	No serious concerns	Direct comparison of MS patients vs. matched controls; outcome directly measured	0
Imprecision	No serious concerns	Narrow CI excluding null (1.57–1.85); large sample size (*n* > 900,000)	0
Publication Bias	Undetected	Trim-and-fill imputed 0 studies; funnel plot symmetric; no evidence of missing studies	0
Starting Certainty	LOW	Observational studies	
Final Certainty	LOW		-1

Evidence certainty rated using the Grading of Recommendations Assessment, Development and Evaluation (GRADE) framework, starting from low certainty for observational evidence. One downgrade was applied for risk of bias due to incomplete confounder adjustment in several studies and reliance on administrative diagnostic codes without validation. No downgrade was applied for publication bias as trim-and-fill analysis imputed zero missing studies and the funnel plot showed reasonable symmetry; final GRADE certainty: LOW

## Discussion

This systematic review and meta-analysis of seven cohort studies involving over 900,000 participants demonstrates that patients with MS have a 70% increased risk of myocardial infarction compared to individuals without MS (RR 1.70, 95% CI: 1.57–1.85). This finding was robust across sensitivity analyses, with leave-one-out analysis showing stable effect estimates (RR range 1.69–1.76) regardless of which study was excluded. The 95% prediction interval (1.60–1.81) indicates that future studies conducted in diverse settings would be expected to demonstrate increased MI risk in MS populations, reinforcing the clinical significance of these findings.

### Comparison with existing literature

Our pooled estimate is consistent with previous meta-analyses examining cardiovascular outcomes in MS. Rapp et al. reported a pooled hazard ratio of 1.6 (95% CI: 1.2-2.0) for MI in MS based on nine longitudinal studies, though with considerable heterogeneity (I²=86%) [7]. Tavallaei et al. reported a pooled OR of 1.41 [8]. Most recently, Ketata and Ellouz reported an IRR of 1.4 (95% CI: 1.0-1.9) for MI in MS, with the important observation that cardiovascular risk was highest within the first 10 years following MS diagnosis [9]. Our estimate of RR 1.70 aligns closely with these prior findings while benefiting from inclusion of newer cohort studies published through 2025 and resolution of substantial heterogeneity through exclusion of an overlapping cohort. The temporal pattern identified by Ketata and Ellouz, elevated risk particularly in the early disease course, has implications for clinical practice, suggesting that cardiovascular surveillance may be most important in the years immediately following MS diagnosis.

Contextualizing our findings within the broader landscape of autoimmune disease-associated cardiovascular risk is informative. A meta-analysis of rheumatoid arthritis demonstrated an MI relative risk of 1.68 (95% CI: 1.40–2.03) [38], while systemic lupus erythematosus carries substantially higher risk (RR 2.92, 95% CI: 2.45–3.48) [39]. Psoriasis, another inflammation-driven condition increasingly recognized as warranting cardiovascular surveillance, shows more modest MI risk elevation (RR 1.17, 95% CI: 1.11–1.24) [40]. Our MS-MI estimate of 1.70 is remarkably similar to RA, and substantially exceeds psoriasis, notable because both RA and psoriasis are already included in cardiovascular risk guidelines recommending enhanced screening. This comparison supports the case for systematic cardiovascular risk assessment in MS populations.

The consistency of our findings with individual high-quality cohort studies strengthens confidence in the results. Marrie et al. reported an adjusted HR of 1.63 (95% CI: 1.43–1.87) in a Canadian cohort of 14,565 MS patients, demonstrating that traditional cardiovascular risk factors do not fully explain the elevated MI incidence [23]. The Korean nationwide cohort study by Cho et al. found an HR of 2.61 (95% CI: 1.73–3.94) [35], remarkably consistent with our Asia-Pacific subgroup estimate (RR 2.61), providing external validation and suggesting potential geographic or ethnic variation in MS-associated cardiovascular risk.

### Mechanistic considerations

The pathophysiological mechanisms underlying elevated cardiovascular risk in MS are multifactorial and extend beyond traditional risk factors. Our finding that adjusted and unadjusted effect estimates were similar (RR 1.88 vs. 1.66, *p* = 0.45) supports the hypothesis that MS-specific pathways contribute independently to cardiovascular risk. Chronic systemic inflammation, mediated by pro-inflammatory cytokines including IL-1β, IL-6, and TNF-α, promotes endothelial dysfunction and accelerates atherosclerosis in autoimmune conditions [41]. The CANTOS trial demonstrated that blocking IL-1β reduced cardiovascular events, providing proof-of-concept that inflammation causally contributes to atherosclerotic disease. A recent meta-analysis of 14 studies confirmed that MS patients exhibit significantly impaired vascular function (SMD 0.56, *p* = 0.02) and increased arterial stiffness (SMD 0.78, *p* = 0.008) compared to controls [42], providing direct evidence of vascular pathology as a mechanistic pathway.

Cardiac autonomic dysfunction affects approximately 42% of MS patients and manifests as reduced heart rate variability, impaired baroreflex sensitivity, and increased susceptibility to arrhythmias [43]. This autonomic impairment has been linked to increased risk of MI, stroke, and heart failure in large cohort studies, representing another MS-specific pathway to cardiovascular events. Furthermore, MS-related disability promotes sedentary behavior, with meta-analytic evidence demonstrating that MS patients engage in significantly more sedentary time than the general population (SMD 0.27, *p* = 0.03), with median daily sitting time approximately double that of controls [44]. This physical inactivity contributes to metabolic derangements that accelerate atherosclerosis independently of inflammatory processes.

Disease-modifying therapies may also influence cardiovascular risk. A pooled analysis of 15 randomized controlled trials demonstrated that sphingosine-1-phosphate receptor modulators (fingolimod, siponimod, ozanimod) significantly increase cardiovascular adverse events (RR 2.21, 95% CI: 1.58–3.10), including bradyarrhythmias and hypertension [45]. Given the increasing use of these agents as first-line therapy, DMT-related cardiovascular effects may contribute to the elevated risk observed in MS populations, highlighting the importance of cardiovascular monitoring during treatment. However, the included studies span decades (1977–2017), during which DMT availability varied considerably, limiting the ability to attribute the observed MI excess specifically to DMT effects.

### Heterogeneity considerations

The moderate heterogeneity observed in our analysis (I²=40.3%) is within acceptable limits for meta-analysis of observational studies. Methodological guidance emphasizes that heterogeneity in meta-analysis should be expected rather than viewed as a limitation requiring study exclusion [46, 47]. Importantly, exclusion of one study with overlapping population (Capkun 2015, which used the same Department of Defense database as Persson 2020 DOD) resolved the substantial heterogeneity (from I²=71.6% to 40.3%) that would otherwise have been present. Our subgroup analyses explored potential sources of remaining variation, revealing a trend toward geographic differences (*p* = 0.066), with higher effect estimates in Asia-Pacific (RR 2.61) compared to European studies (RR 1.49). This variation may reflect differences in baseline cardiovascular disease rates, MS phenotypes across ethnic groups, healthcare access affecting outcome ascertainment, or regional variation in vascular comorbidity prevalence [48]. The narrow prediction interval (1.60–1.81) demonstrates that despite moderate heterogeneity, the clinical conclusion of elevated MI risk in MS remains consistent.

### Clinical implications

While our findings suggest clinically meaningful cardiovascular risk elevation in MS, several caveats apply to clinical translation. No MS-specific cardiovascular screening guidelines currently exist, and our evidence, graded as low certainty, requires cautious interpretation. The European League Against Rheumatism (EULAR) provides a model framework for rheumatoid arthritis, recommending cardiovascular risk assessment at least every five years and application of a 1.5× multiplication factor to traditional risk scores [49]. However, the RA cardiovascular literature is supported by a substantially larger and more consistent evidence base than currently exists for MS. Updated EULAR recommendations extend cardiovascular surveillance principles to multiple autoimmune conditions, emphasizing aggressive management of modifiable risk factors and disease activity control [50]. An international workshop endorsed by ECTRIMS and the National MS Society identified hypertension, hyperlipidemia, and diabetes as priority comorbidities requiring research attention in MS [51], though this provided research recommendations rather than clinical guidelines. Recent expert reviews confirm that optimal screening intervals remain unknown and clinical trials testing cardiovascular prevention interventions in MS are needed before definitive screening recommendations can be made [52]. In the interim, clinicians should be aware of the potential for elevated cardiovascular risk in MS patients and consider standard cardiovascular risk factor assessment and management.

### Strengths and limitations

This systematic review has several strengths. We adhered to PRISMA 2020 [14] and MOOSE [15] reporting guidelines for transparent methodology. We incorporated newer cohort studies published through 2025, providing a contemporary estimate of MI risk. Careful assessment of population overlap led to exclusion of one study using the same database as another included cohort, substantially reducing heterogeneity. Integration of Mendelian randomization evidence [13] provided complementary genetic data, though the small effect sizes warrant cautious interpretation. Application of the Hartung-Knapp-Sidik-Jonkman adjustment provided more appropriate confidence intervals given the number of included studies.

Several limitations merit consideration. First, as a meta-analysis of observational studies, residual confounding cannot be excluded despite statistical adjustment. Simulation studies demonstrate that unmeasured confounders can generate spurious associations of the magnitude observed in epidemiological research [53]. Second, surveillance bias may partly explain the observed association: MS patients interact more frequently with the healthcare system than the general population, potentially increasing the likelihood of MI detection. Third, most included studies relied on administrative diagnostic codes for MI ascertainment without clinical validation. However, validation studies indicate hospitalization-based ICD codes for MI have positive predictive values exceeding 93% [54], supporting reasonable outcome accuracy. Fourth, the limited number of studies (k = 7) precluded robust assessment of publication bias using Egger’s test, limited statistical power for meta-regression to formally explore sources of heterogeneity, and prevented Trial Sequential Analysis to evaluate whether the accumulated evidence is sufficient to support firm conclusions. Fifth, we could not assess whether specific MS subtypes, disease severity (EDSS), or disability levels differentially influence cardiovascular risk due to inconsistent reporting across primary studies. Sixth, the studies spanned several decades during which disease-modifying therapy availability changed substantially, limiting our ability to disentangle intrinsic MS-related risk from potential iatrogenic effects. Finally, the MR evidence, while statistically significant, showed small effect sizes (2–3% increased risk) that contrast with the larger observational estimate, suggesting that unmeasured confounding or other biases may partly explain the observational association.

### Future research directions

Several research priorities emerge from this analysis. Prospective cohort studies with standardized cardiovascular outcome ascertainment and comprehensive phenotyping of MS populations are needed to clarify risk stratification. Clinical trials testing whether aggressive cardiovascular risk factor management improves outcomes in MS patients are essential to translate epidemiological findings into clinical practice. Development of MS-specific cardiovascular risk prediction tools, potentially incorporating machine learning approaches analyzing electronic health records, imaging biomarkers, and inflammatory markers, represents a promising avenue, though current AI-based models require rigorous external validation before clinical deployment [55]. Finally, mechanistic studies examining the contribution of specific inflammatory pathways, autonomic dysfunction, and treatment effects to cardiovascular risk could identify novel therapeutic targets.

## Conclusion

In conclusion, this systematic review and meta-analysis demonstrates that MS patients face a 70% increased risk of myocardial infarction compared to individuals without MS (RR 1.70, 95% CI: 1.57–1.85), an association that is robust across sensitivity analyses, consistent across geographic regions, and comparable to the cardiovascular risk elevation observed in rheumatoid arthritis, a condition for which cardiovascular screening guidelines already exist. Mendelian randomization evidence provides suggestive but not definitive support for a causal relationship given the small genetic effect sizes. These findings support the need for heightened cardiovascular risk awareness among clinicians caring for MS patients and the development of evidence-based guidelines for cardiovascular screening and prevention in this population.

## Supplementary Information

Supplementary Material 1.

Supplementary Material 2.

## Data Availability

Data is provided within the manuscript or supplementary information files.

## Abbreviations

AF: Atrial fibrillation
CAD: Coronary artery disease
CANTOS: Canakinumab anti–inflammatory thrombosis outcome study
CI: Confidence interval
CPRD: Clinical practice research datalink
CVD: Cardiovascular disease
DALY: Disability–adjusted life year
DMT: Disease–modifying therapy
DNRP: Danish national registry of patients
ECTRIMS: European committee for treatment and research in multiple sclerosis
ERR: Event rate ratio
EULAR: European league against rheumatism
GRADE: Grading of recommendations assessment, development and evaluation
HR: Hazard ratio
ICD: International classification of diseases
IL: Interleukin
IRR: Incidence rate ratio
IVW: Inverse–variance weighted
KNHIS: Korean national health insurance service
MI: Myocardial infarction
MOOSE: Meta–analysis of observational studies in epidemiology
MR: Mendelian randomization
MS: Multiple sclerosis
NOS: Newcastle–Ottawa scale
OR: Odds ratio
RA: Rheumatoid arthritis
REML: Restricted maximum likelihood
RR: Relative risk
SLE: Systemic lupus erythematosus
SMD: Standardized mean difference
SNP: Single nucleotide polymorphism
TNF: Tumor necrosis factor
VTE: Venous thromboembolism
