# Supplementary material for: Risk of myocardial infarction in patients with multiple sclerosis: a systematic review and meta-analysis
Source: BMC Neurol. 2026 Apr 13;26:338. doi: 10.1186/s12883-026-04882-w (PMC13188473; doi:10.1186/s12883-026-04882-w)
Supplement: Supplementary file 1 — Supplementary Material 1. [file 12883_2026_4882_MOESM1_ESM.docx]

**Risk of Myocardial Infarction in Patients with Multiple Sclerosis: A Systematic Review and Meta-Analysis**

**Supplementary Tables**

**Supplementary Table S1. Search Strategy**

**Supplementary Table S2. Secondary Outcomes Meta-Analysis Results**

**Supplementary Table S3. Risk of Bias Assessment**

**Supplementary Table S4. Mendelian Randomization Evidence**

**Supplementary Table S5. Leave-One-Out Sensitivity Analysis**

**Supplementary Table S6. Excluded Studies with Reasons**

**Supplementary Figures**

**Figure S1.** **MI subgroup analysis by effect measure (HR vs IRR vs ERR).**

**Figure S2.** **Funnel plot for MI.**

**Figure S3.** **Funnel plot with trim-and-fill for MI.**

**Figure S4.** **Leave-one-out sensitivity analysis for MI.**

**Figure S5.** **Summary forest plot of pooled effects for MI, stroke, and VTE.**

**Figure S6. MI prevalence (OR studies)**

**Figure S7. Risk of bias assessment**

**SUPPLEMENTARY TABLES**

**Supplementary Table S1. Search Strategy**

| **Database** | **Search strategy (copy-paste single block)** | **Date (search end)** | **Results** |
| --- | --- | --- | --- |
| **PubMed/MEDLINE** | (("multiple sclerosis"[MeSH Terms] OR "multiple sclerosis"[Title/Abstract] OR "MS"[Title/Abstract] OR "demyelinating disease"[Title/Abstract])  AND  ("myocardial infarction"[MeSH Terms] OR "myocardial infarction"[Title/Abstract] OR "heart attack"[Title/Abstract] OR "acute coronary syndrome"[Title/Abstract] OR "cardiovascular diseases"[MeSH Terms] OR "cardiovascular"[Title/Abstract] OR "stroke"[MeSH Terms] OR "stroke"[Title/Abstract] OR "ischemic heart disease"[Title/Abstract])) | **Dec 2025** | **1245** |
| **Embase (embase.com)** | ('multiple sclerosis'/exp OR 'multiple sclerosis':ti,ab OR 'MS':ti,ab OR 'demyelinating disease':ti,ab)  AND  ('myocardial infarction'/exp OR 'myocardial infarction':ti,ab OR 'heart attack':ti,ab OR 'acute coronary syndrome':ti,ab OR 'cardiovascular disease'/exp OR 'cardiovascular':ti,ab OR 'stroke'/exp OR 'stroke':ti,ab OR 'ischemic heart disease':ti,ab) | **Dec 2025** | **1,102** |
| **Scopus** | TITLE-ABS-KEY("multiple sclerosis" OR "MS" OR "demyelinating disease")  AND  TITLE-ABS-KEY("myocardial infarction" OR "heart attack" OR "acute coronary syndrome" OR "cardiovascular" OR "stroke" OR "ischemic heart disease") | **Dec 2025** | 550 |
| **Total from databases** |  |  | **2,897** |
| **Records from other sources** | Reference lists / citation searching / manual searching | **Dec 2025** | **0** |
| **Total records identified** |  |  | **2,897** |
| **Duplicates removed** |  |  | **736** |
| **After deduplication (records screened)** |  |  | **2,161** |

Comprehensive search strategies across PubMed/MEDLINE, Embase, and Scopus combining MeSH terms and keywords for multiple sclerosis and cardiovascular outcomes, conducted through December 2025, yielding 2,897 total records. Additional records identified through reference lists, citation searching, and manual searching of relevant journals.

**Supplementary Table S2. Secondary Outcomes Meta-Analysis Results**

**Supplementary Table S2. Secondary Outcomes Meta-Analysis Results**

| **Outcome** | **k** | **MS (n)** | **Controls (n)** | **Pooled RR (95% CI)** | **I² (%)** | **p-value** |
| --- | --- | --- | --- | --- | --- | --- |
| Myocardial Infarction | 7 | 97,996 | 824,497 | 1.70 (1.57-1.85) | 40.3 | <0.0001 |
| Stroke | 3 | 18,734 | 185,440 | 1.66 (1.01-2.73) | 33.4 | 0.049 |
| Venous Thromboembolism | 3 | 18,734 | 185,440 | 1.93 (0.93-4.01) | 88.2 | 0.061 |
| MI Prevalence (OR)* | 2 | — | — | 0.73 (0.64-0.82) | 0.0 | <0.0001 |

*Cross-sectional studies reporting odds ratios; analyzed separately from incidence studies

All pooled estimates derived using random-effects meta-analysis with restricted maximum likelihood estimation and Hartung-Knapp-Sidik-Jonkman confidence interval adjustment. *"†MI Prevalence (OR) derived using fixed-effects meta-analysis given I²=0.0%; all other estimates derived using random-effects meta-analysis.* k = number of cohorts; RR = relative risk; CI = confidence interval; I² = between-study heterogeneity.

**Supplementary Table S3. Risk of Bias Assessment**

| **Study** | **Selection (max 4)** | **Comparability (max 2)** | **Outcome (max 3)** | **Total (max 9)** | **Judgment** |
| --- | --- | --- | --- | --- | --- |
| Allen 2008 | 4 | 2 | 2 | 8 | Low |
| Christiansen 2010 | 3 | 2 | 3 | 8 | Low |
| Herial 2012 | — | — | — | 6 | Moderate |
| Capkun 2014 | — | — | — | 5 | Moderate |
| Capkun 2015 | 3 | 1 | 2 | 6 | Moderate |
| Castelo-Branco 2020 | 4 | 1 | 3 | 8 | Low |
| Cho 2022 | 3 | 2 | 2 | 7 | Low |
| Persson 2020 (DOD) | 3 | 2 | 2 | 7 | Low |
| Persson 2020 (CPRD) | 3 | 2 | 2 | 7 | Low |
| Marrie 2019 | 4 | 2 | 3 | 9 | Low |
| Chou 2020 | 4 | 2 | 2 | 8 | Low |
| Franklin 2014 | 2 | 1 | 1 | 4 | Moderate |
| Kresa-Reahl 2017 | 3 | 1 | 2 | 6 | Moderate |
| Benjaminsen 2021 | — | — | — | 8 | Low |
| Lo 2021 | — | — | — | 9 | Low |
| Bezzini 2022 | — | — | — | 7 | Low |
| Cabreira 2020 | 2 | 1 | 3 | 6 | Moderate |
| Giallafos 2016 | — | — | — | 4 | High |
| Goodman 2014 | — | — | — | 5 | Moderate |
| Hemida 2025 | — | — | — | 7 | Low |

Cohort studies assessed using the Newcastle-Ottawa Scale (NOS; maximum 9 stars): low risk ≥7, moderate risk 4–6, high risk ≤3. Cross-sectional and descriptive studies (denoted "—") assessed using the JBI checklist with scores converted to the 9-point scale. Of 20 cohorts, 12 (60%) low risk, 7 (35%) moderate risk, and 1 (5%) high risk; mean NOS 6.8/9. Two reviewers independently assessed each study; disagreements resolved by consensus.

**Supplementary Table S4. Mendelian Randomization Evidence (Yang et al., 2022)**

| **Exposure** | **Outcome** | **SNPs** | **Method** | **OR (95% CI)** | **p-value** | **Pleiotropy p** | **Heterogeneity p** |
| --- | --- | --- | --- | --- | --- | --- | --- |
| MS liability | CAD | 68 | IVW | 1.02 (1.00-1.04) | 0.03 | 0.167 | 0.132 |
| MS liability | MI | 68 | IVW | 1.03 (1.00-1.06) | 0.01 | 0.158 | 0.517 |
| MS liability | Heart Failure | 68 | IVW | 1.02 (1.00-1.04) | 0.02 | 0.117 | 0.005 |
| MS liability | All Stroke | 68 | IVW | 1.02 (1.00-1.05) | 0.02 | 0.525 | 0.027 |
| MS liability | Ischemic Stroke | 68 | IVW | 1.02 (1.00-1.05) | 0.04 | 0.844 | 0.020 |
| MS liability | AF | 68 | IVW | 1.00 (0.98-1.01) | 0.92 | 0.996 | 0.002 |

Data sourced from Yang et al. (2022) using 68 genome-wide significant SNPs as instruments for MS genetic liability. All estimates derived by inverse-variance weighted (IVW) method; pleiotropy assessed by MR-Egger intercept test (p >0.05 indicates no directional pleiotropy). The small magnitude of genetic effect sizes (OR 1.02–1.03) should be interpreted cautiously and does not imply equivalence with the larger observational estimates. OR = odds ratio; CAD = coronary artery disease; MI = myocardial infarction; AF = atrial fibrillation.

**Supplementary Table S5. Leave-One-Out Sensitivity Analysis**

| **Study Omitted** | **Pooled RR (95% CI)** | **I² (%)** | **95% Prediction Interval** |
| --- | --- | --- | --- |
| Capkun 2014 | 1.75 (1.35-2.25) | 50.2 | 1.09-2.79 |
| Castelo-Branco 2020 | 1.71 (1.57-1.86) | 35.9 | 1.60-1.83 |
| Cho 2022 | 1.69 (1.57-1.82) | 14.4 | 1.58-1.81 |
| Christiansen 2010 | 1.70 (1.54-1.87) | 49.3 | 1.59-1.82 |
| Marrie 2019 | 1.76 (1.39-2.24) | 47.9 | 1.18-2.64 |
| Persson 2020 (CPRD) | 1.71 (1.56-1.87) | 44.3 | 1.59-1.82 |
| Persson 2020 (DOD) | 1.69 (1.55-1.84) | 36.6 | 1.58-1.81 |
| **Overall** | **1.70 (1.57-1.85)** | **40.3** | **1.60-1.81** |

Each row represents the pooled relative risk obtained after sequentially omitting the indicated study from the primary meta-analysis (k = 7 cohorts). Stability of estimates across all iterations (RR range 1.69–1.76) confirms that no single study disproportionately drives the overall result. The 95% prediction interval represents the expected range of true effects in a new comparable study. RR = relative risk; CI = confidence interval.

**Supplementary Table S6. Excluded Studies with Reasons**

| **Study** | **Year** | **Reason for Exclusion** |
| --- | --- | --- |
| Tanaka | 1985 | Wrong outcome (immunological study) |
| Clarke (STAR_MS) | 2012 | Wrong outcome (diagnostic study) |
| Brnabic | 2024 | Wrong comparison (DMT comparison within MS) |
| Yang | 2022 | Different design (MR study - analyzed separately) |
| Manco | 2024 | Wrong population (vascular MCI, not MS) |
| Chang | 2018 | Case report |
| Pangan Lo | 2021 | Wrong outcome (QoL study) |
| Ragonese | 2017 | Drug-specific (mitoxantrone cardiotoxicity) |
| France-Ratcliffe | 2024 | Within-MS comparison (vitamin D levels) |

Studies excluded at full-text review are listed with the primary reason for exclusion per predefined eligibility criteria. The Yang 2022 Mendelian randomization study was not excluded on methodological grounds but was analyzed separately as supporting causal evidence rather than pooled with observational incidence estimates. MR = Mendelian randomization; DMT = disease-modifying therapy; QoL = quality of life.

**Supplementary Figures**

**Figure S1.** **MI subgroup analysis by effect measure (HR vs IRR vs ERR).**


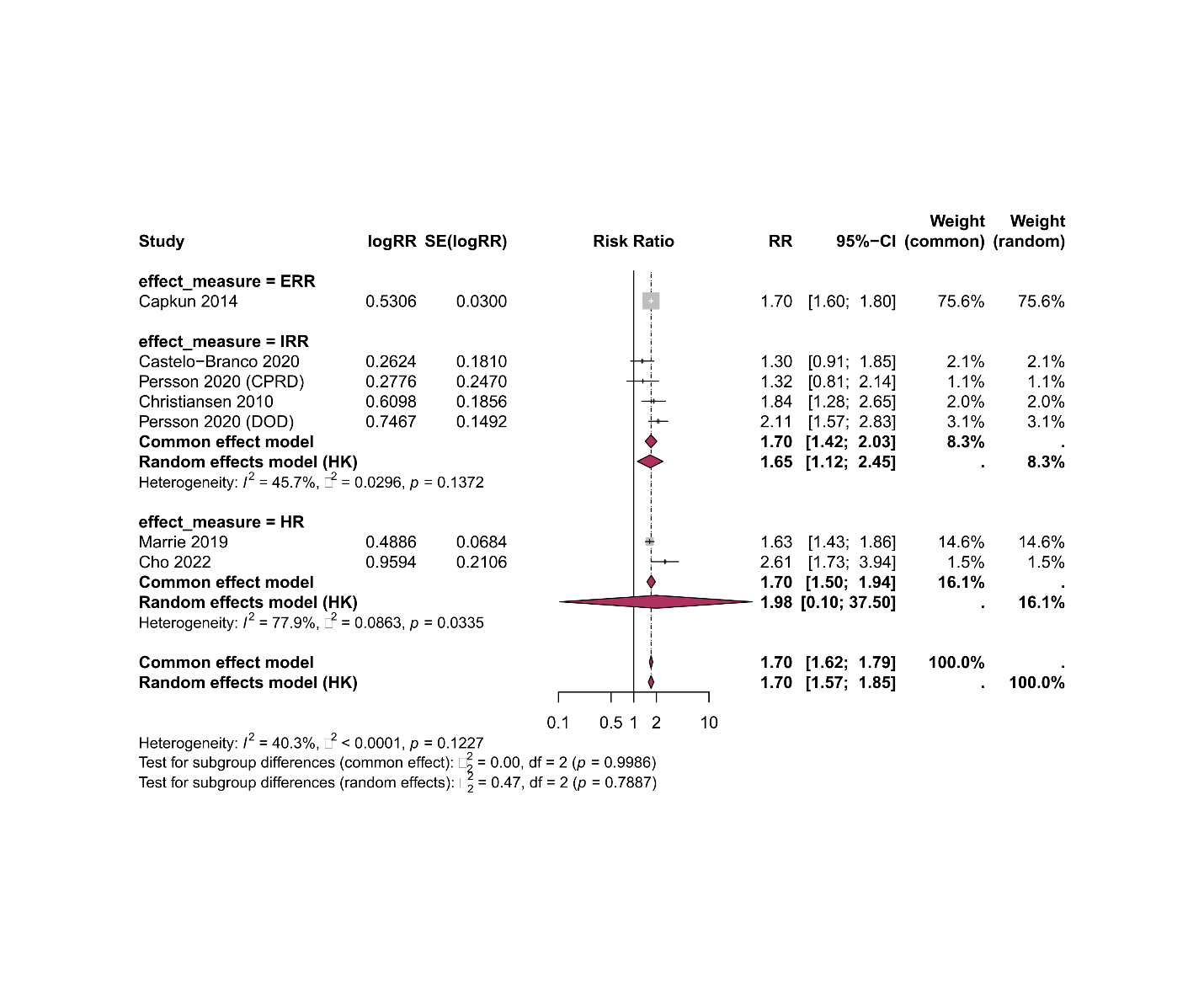


Forest plot displaying pooled relative risk estimates stratified by effect measure type: hazard ratio (HR, k=2), incidence rate ratio (IRR, k=4), and event rate ratio (ERR, k=1). The wide confidence interval for HR studies (95% CI: 0.10–37.50) reflects the Hartung-Knapp adjustment behavior with only two studies and should be interpreted with caution. No significant subgroup difference was detected (p=0.79).

**Figure S2.** **Funnel plot for MI.**


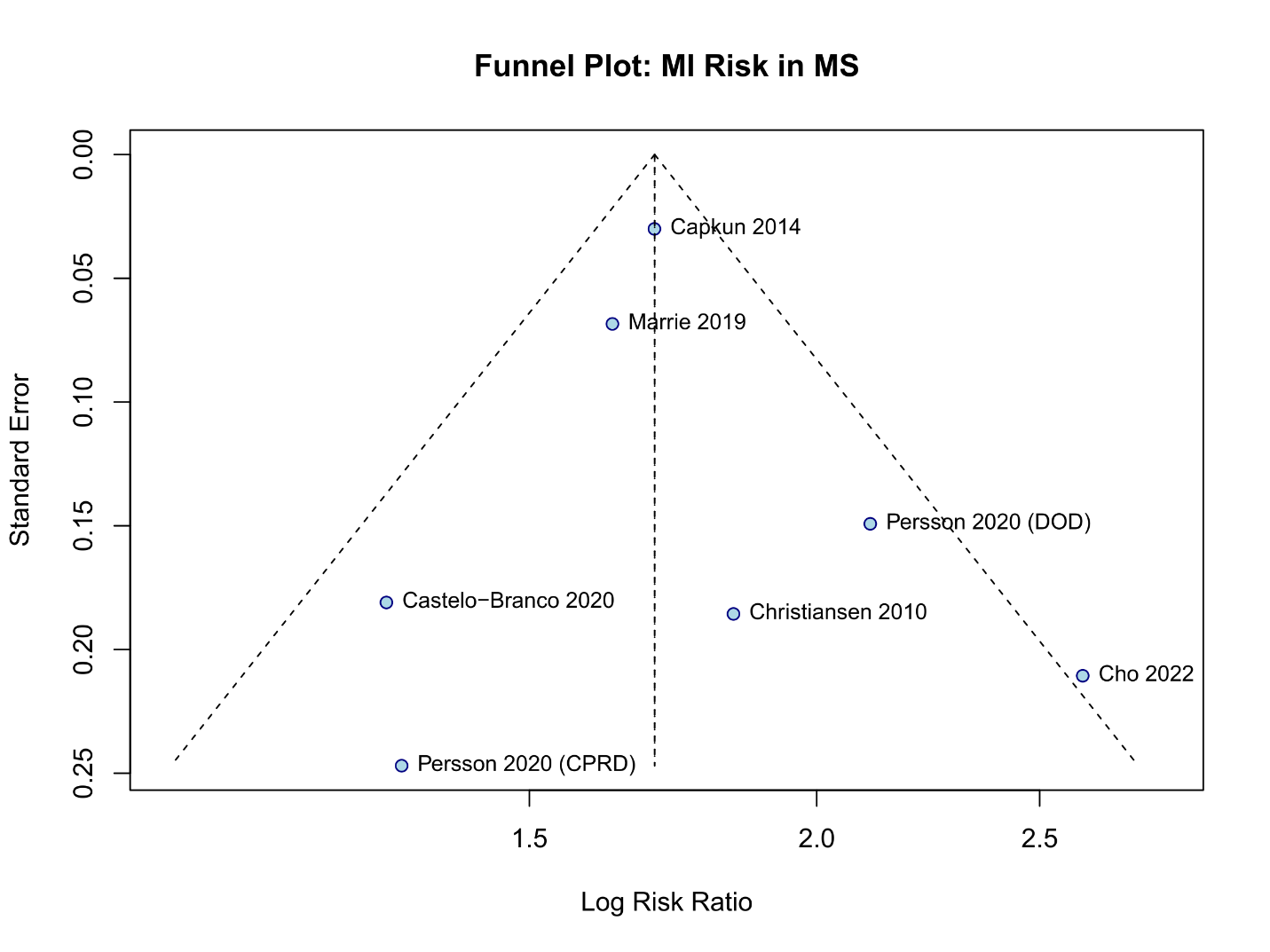


Funnel plot of standard error against log relative risk for the seven cohorts included in the primary meta-analysis. Visual inspection revealed no marked asymmetry. Egger's formal test was not performed due to insufficient studies (k<10), as asymmetry tests are considered unreliable below this threshold. The plot is provided for descriptive purposes only.

**Figure S3.** **Funnel plot with trim-and-fill for MI**


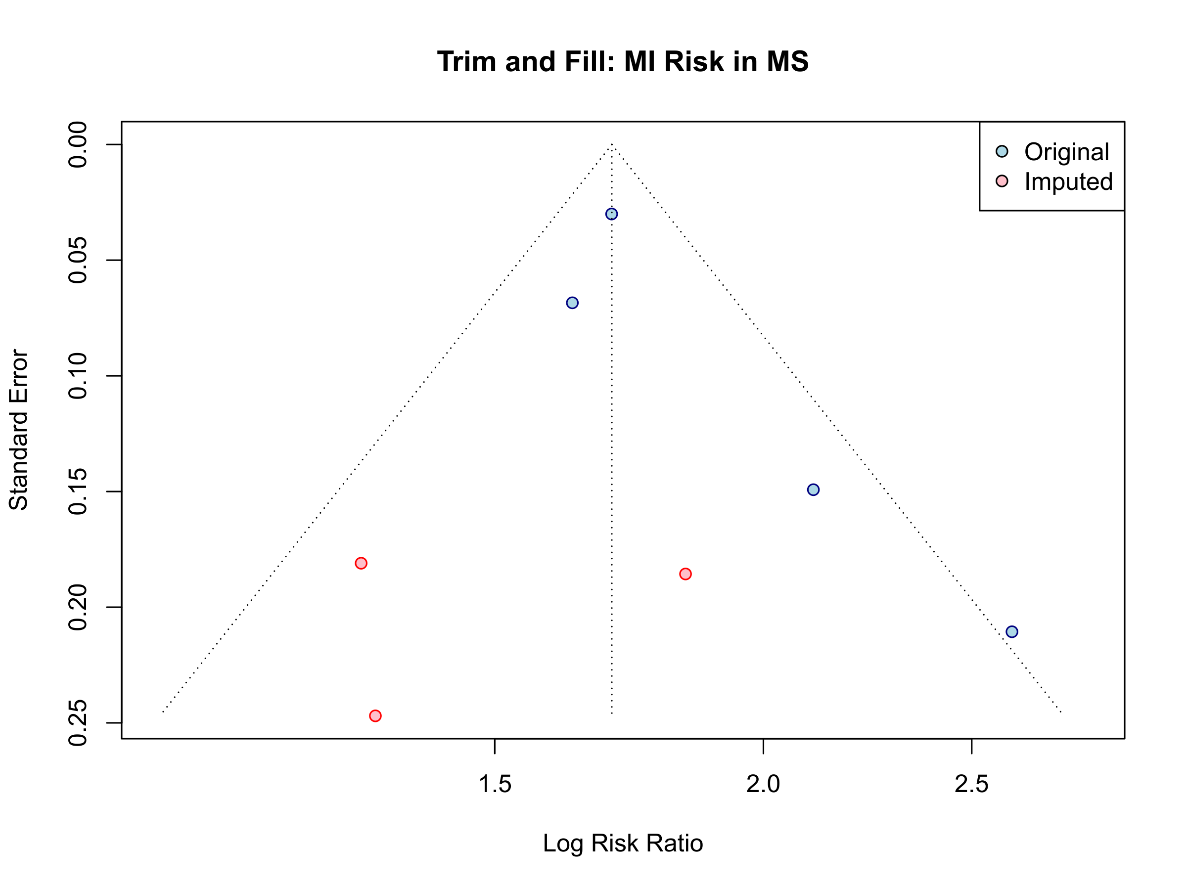


Trim-and-fill analysis (Duval and Tweedie method) applied to assess the potential impact of unpublished studies on the pooled estimate. Zero studies were imputed, and the adjusted pooled estimate remained unchanged at RR 1.70 (95% CI: 1.57–1.85), indicating no evidence of publication bias by this method.

**Figure S4.** **Leave-one-out sensitivity analysis for MI.**


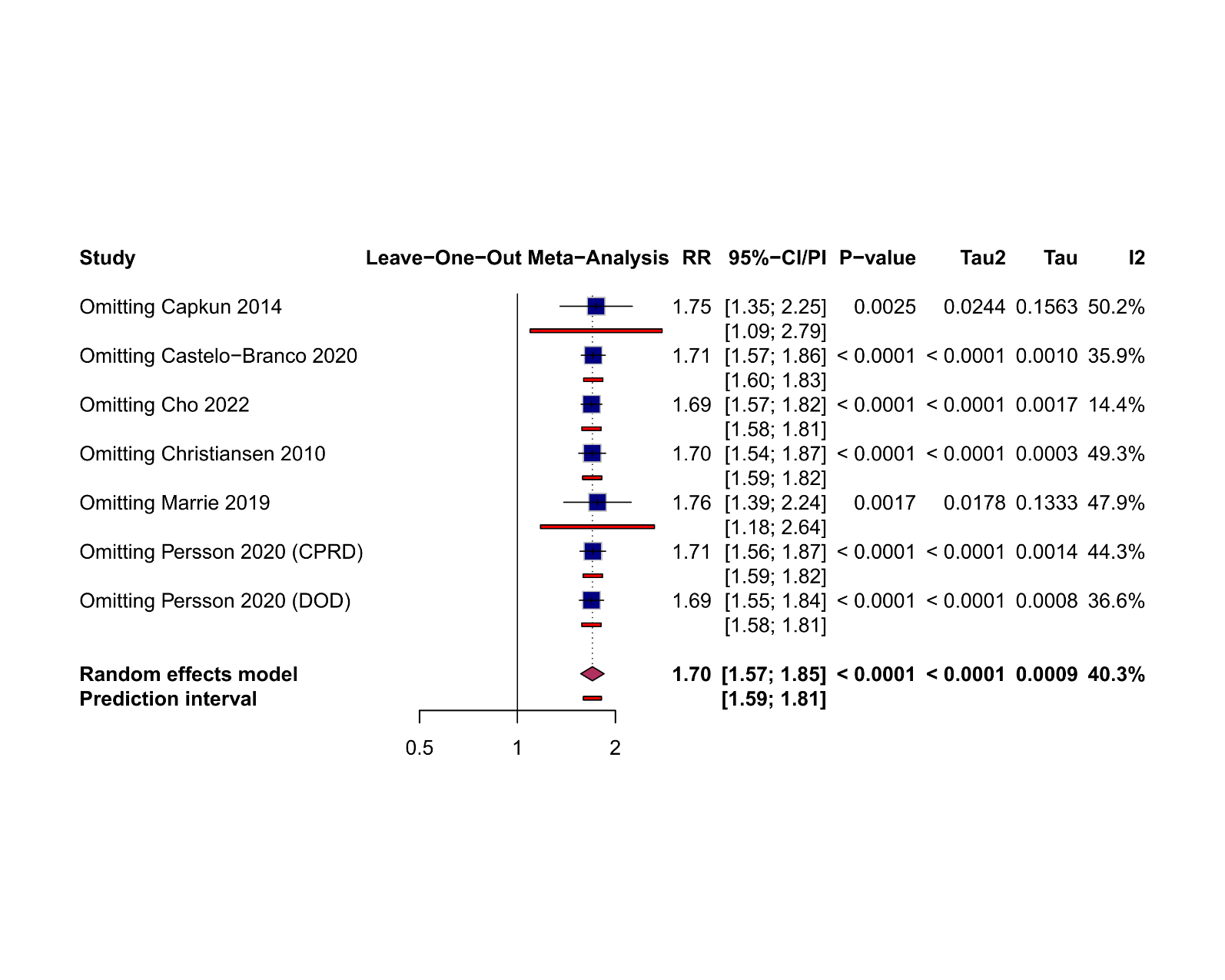


Each point represents the pooled relative risk with 95% confidence interval after sequential omission of one study. The overall pooled estimate (dashed vertical line, RR 1.70) is shown for reference. The narrow range of estimates across all iterations (RR 1.69–1.76) confirms robustness of the primary finding to individual study influence.

**Figure S5.** **Summary forest plot of pooled effects for MI, stroke, and VTE.**


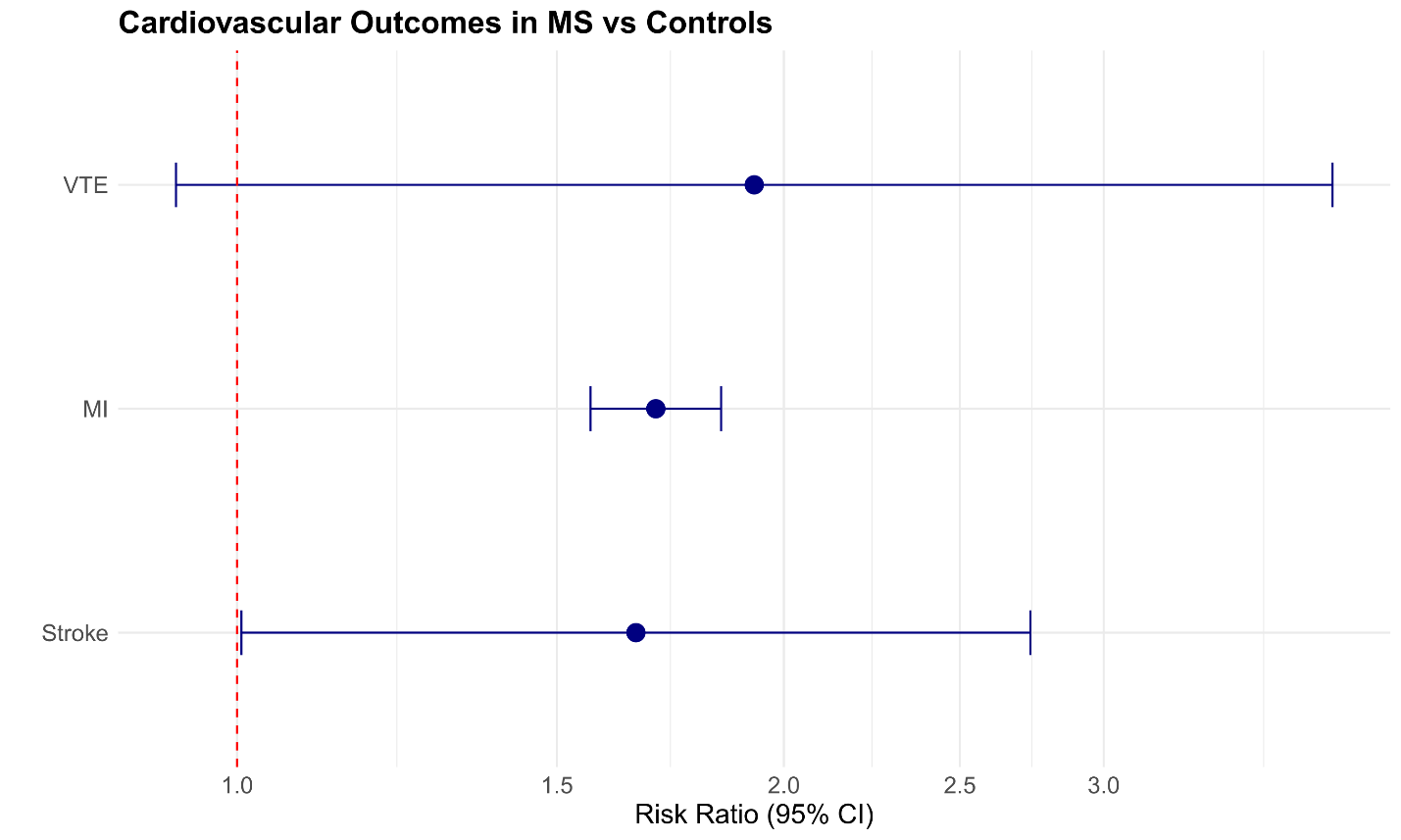


Summary forest plot displaying pooled relative risk estimates for all three prespecified outcomes: myocardial infarction (k=7), stroke (k=3), and venous thromboembolism (k=3). All analyses used random-effects meta-analysis with Hartung-Knapp-Sidik-Jonkman adjustment. The VTE estimate did not reach statistical significance (p=0.061) owing to substantial heterogeneity (I²=88.2%). RR = relative risk; CI = confidence interval; VTE = venous thromboembolism.

**Figure S6. MI prevalence (OR studies)**


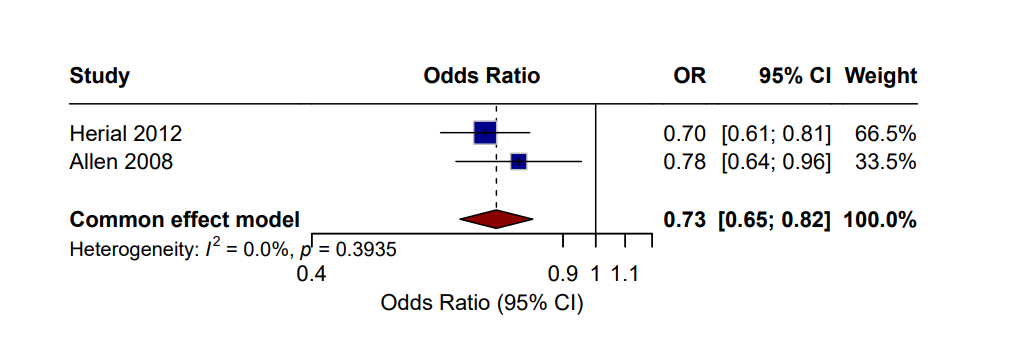


**Abbreviations:** OR, odds ratio; CI, confidence interval; I², inconsistency statistic.

**Notes:** Pooled estimate derived using the common effect model (Mantel-Haenszel method). Heterogeneity was absent (I² = 0.0%, p = 0.3935), supporting model selection. Square size reflects study weight; diamond represents the pooled OR with 95% CI.

**Figure S7. Risk of bias assessment**


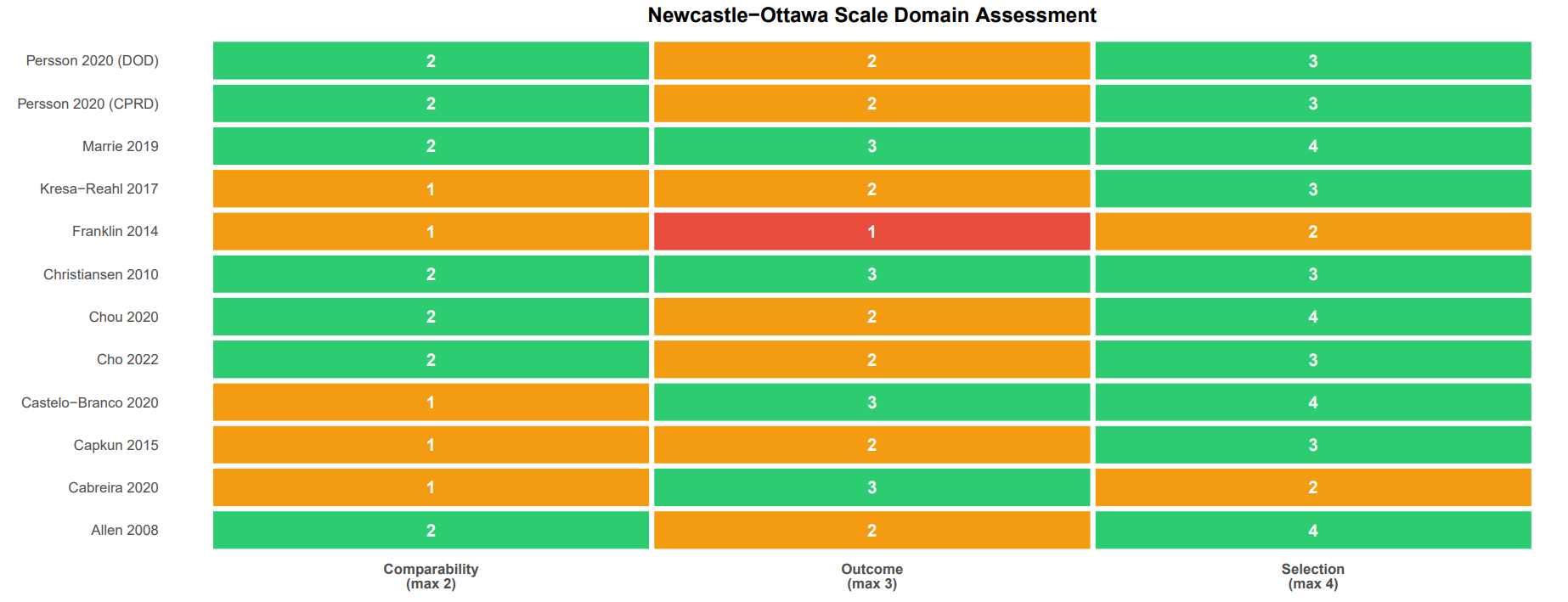


Color-coded domain score plot displaying NOS scores for the 12 cohort and case-control studies assessed using the Newcastle-Ottawa Scale. Three domains are shown: Comparability (maximum 2 stars), Outcome (maximum 3 stars), and Selection (maximum 4 stars). Each cell displays the numerical domain score; color reflects performance level: green indicates higher scores (lower risk of bias), orange indicates intermediate scores (moderate risk), and red indicates lower scores (higher risk of bias). Cross-sectional studies (n=8) were assessed using the JBI checklist and are displayed separately. Among all 20 included cohorts, 12 (60%) were rated low risk of bias (NOS ≥7), 7 (35%) moderate risk (NOS 4–6), and 1 (5%) high risk (NOS ≤3); mean NOS score 6.8/9. The most common limitations were incomplete adjustment for cardiovascular confounders (Comparability domain) and reliance on administrative diagnostic codes without clinical validation (Outcome domain). Two reviewers independently assessed all studies; disagreements were resolved by consensus.
